# Supplementary figures and images for: From complete cross-docking to partners identification and binding sites predictions
Source: PLoS Comput Biol. 2022 Jan 28;18(1):e1009825. doi: 10.1371/journal.pcbi.1009825 (PMC8827487; doi:10.1371/journal.pcbi.1009825)

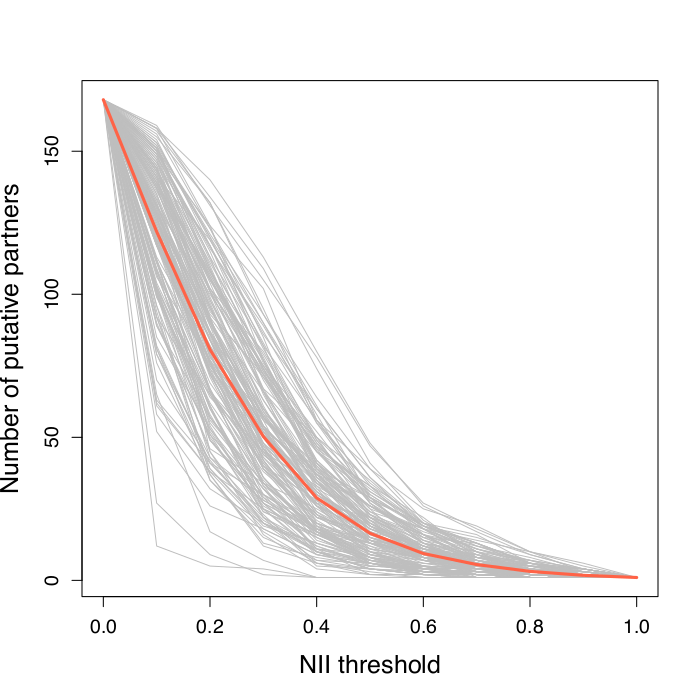

Supplement: S1 Fig — Each grey curve corresponds to a protein from the PPDBv2, and indicates the number of putative partners (y-value) with a NII greater than a threshold (x-value). The red curve shows the average behaviour. (TIF) [file pcbi.1009825.s001.tif]

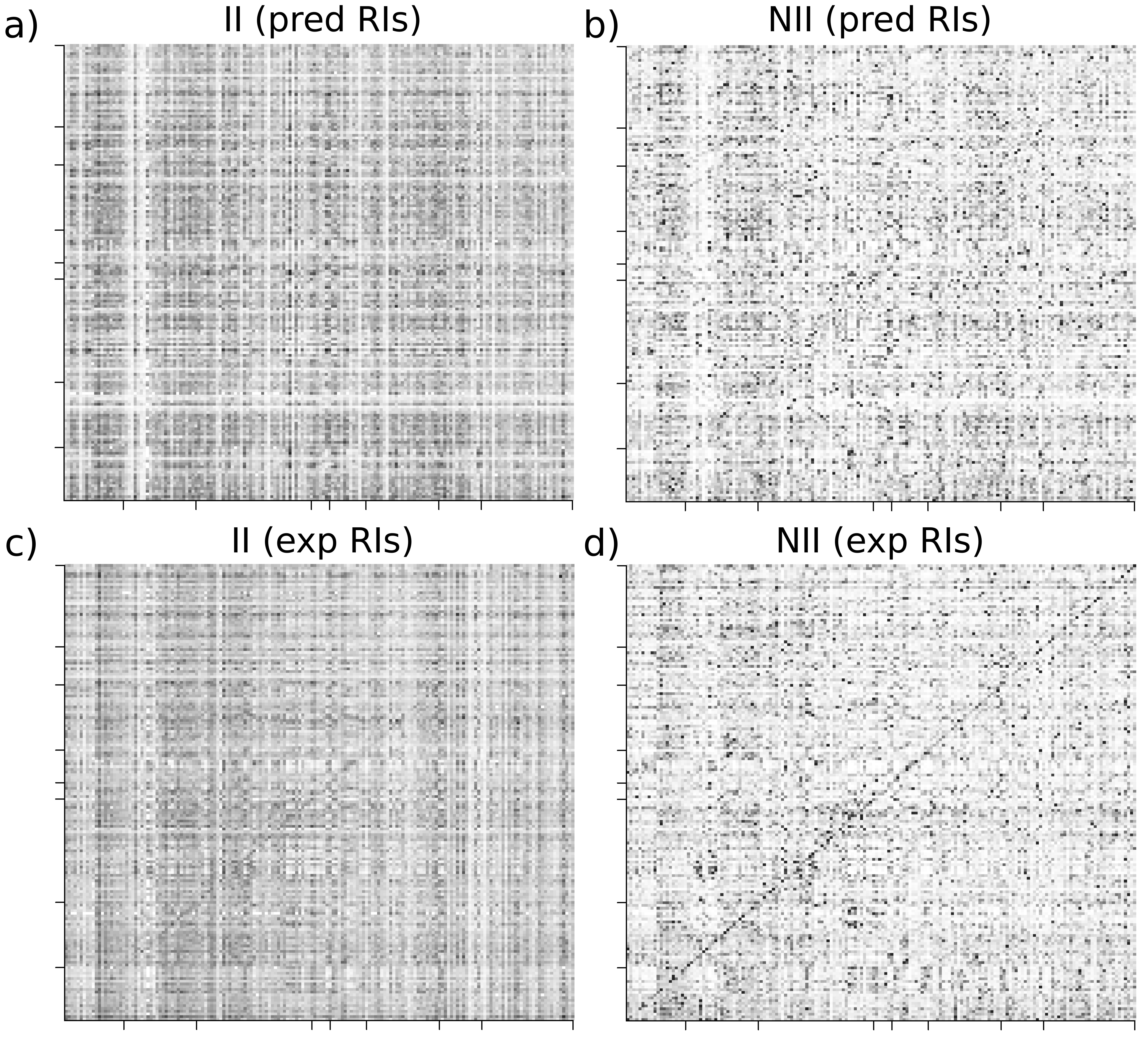

Supplement: S2 Fig — (A-B) Matrices computed using predicted interfaces as references. (C-D) Matrices computed using experimental interfaces as references. The matrices on the left give interaction indices (II) and those on the right the normalized interaction indices (NII). (TIF) [file pcbi.1009825.s002.tif]

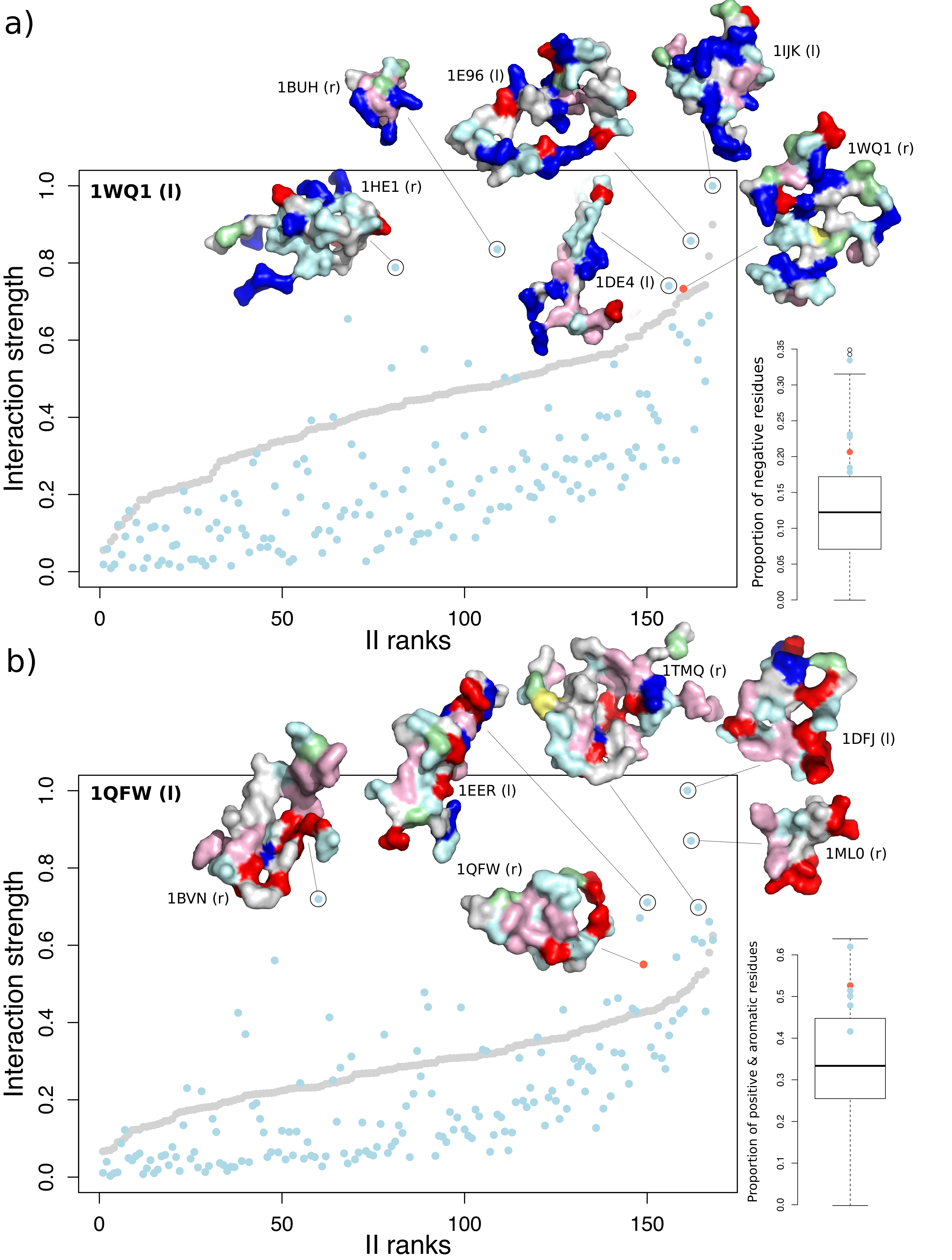

Supplement: S3 Fig — The “interaction strength” is used for plotting pairs with respect to II values (grey) and NII values (blue). The 168 proteins are ordered along the x-axis according to the II ranks they obtained with the protein of interest, and for each position on the x-axis, two points are plotted. The point corresponding to the NII value of the cognate partner is highlighted in red. The II values are scaled between 0 and 1. The predicted RIs for the cognate partner and the top 5 competitors are depicted as surfaces colored by amino acid properties: positive (KR) in blue, negative (DE) in red, polar (HNQST) in cyan, aromatic (FWY) in pink, hydrophobic (AGILMV) in white, cysteine (C) in yellow, and proline (P) in green. The boxplots show the distribution of the proportion of positives (panel a) or negatives and aromatic (panel b) residues in the RIs. The values for the cognate partner and the top 5 competitors are indicated by colored dots. (TIF) [file pcbi.1009825.s003.tif]

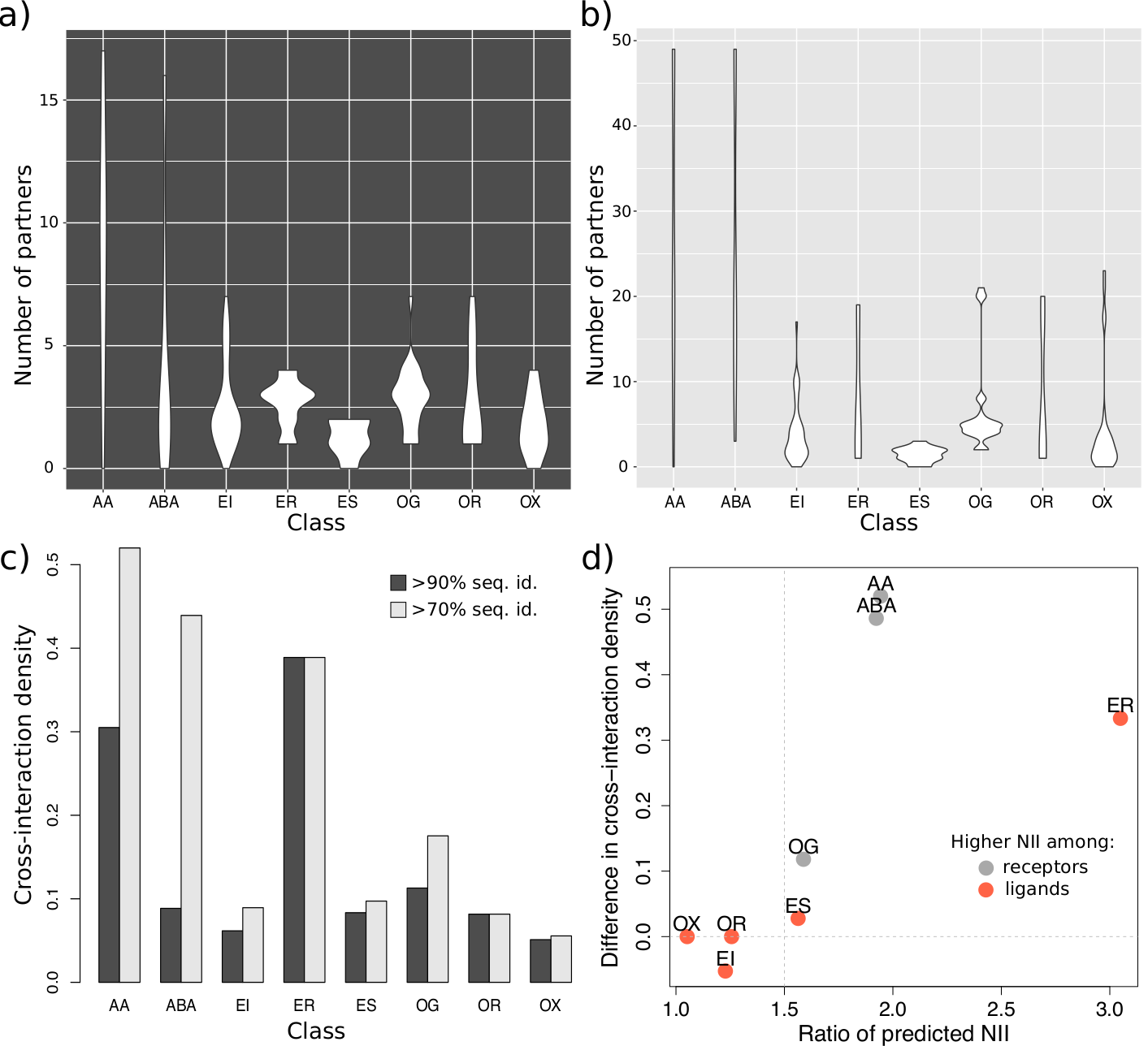

Supplement: S4 Fig — (A-B) Distributions of the number of partners, for each protein within each subset, inferred by homology at 90% (a) and 70% (b) sequence identity levels. (C) Cross-interaction density, defined as the percentage of cells corresponding to a known interaction, within the matrix associated to each subset. The two grey tones indicate the sequence identity level. (D) Agreement between cross-interaction density and predicted NII values. In x-axis are reported the ratios rk=max(∑i,j∈SkNIIRi,Rj∑i,j∈SkNIILi,Lj,∑i,j∈SkNIILi,Lj∑i,j∈SkNIIRi,Rj). For each subset Sk, rk reflects the difference in predicted interaction strengths among the receptors versus the ligands. When the dot is grey, it means the receptors are predicted to interact more with each other, while a red dot indicates that the ligands interact more. In y-axis are reported the different of cross-interactions densities between receptors and ligands, or reciprocally. When the value is positive, it means the tendency observed for the known interactions agrees with that observed for the predictions. For instance, antibodies are predicted to interact with each other twice more than antigens, and there are 50% more known interactions between them. Known interactions were determined with a sequence identity level of 70%. (TIF) [file pcbi.1009825.s004.tif]

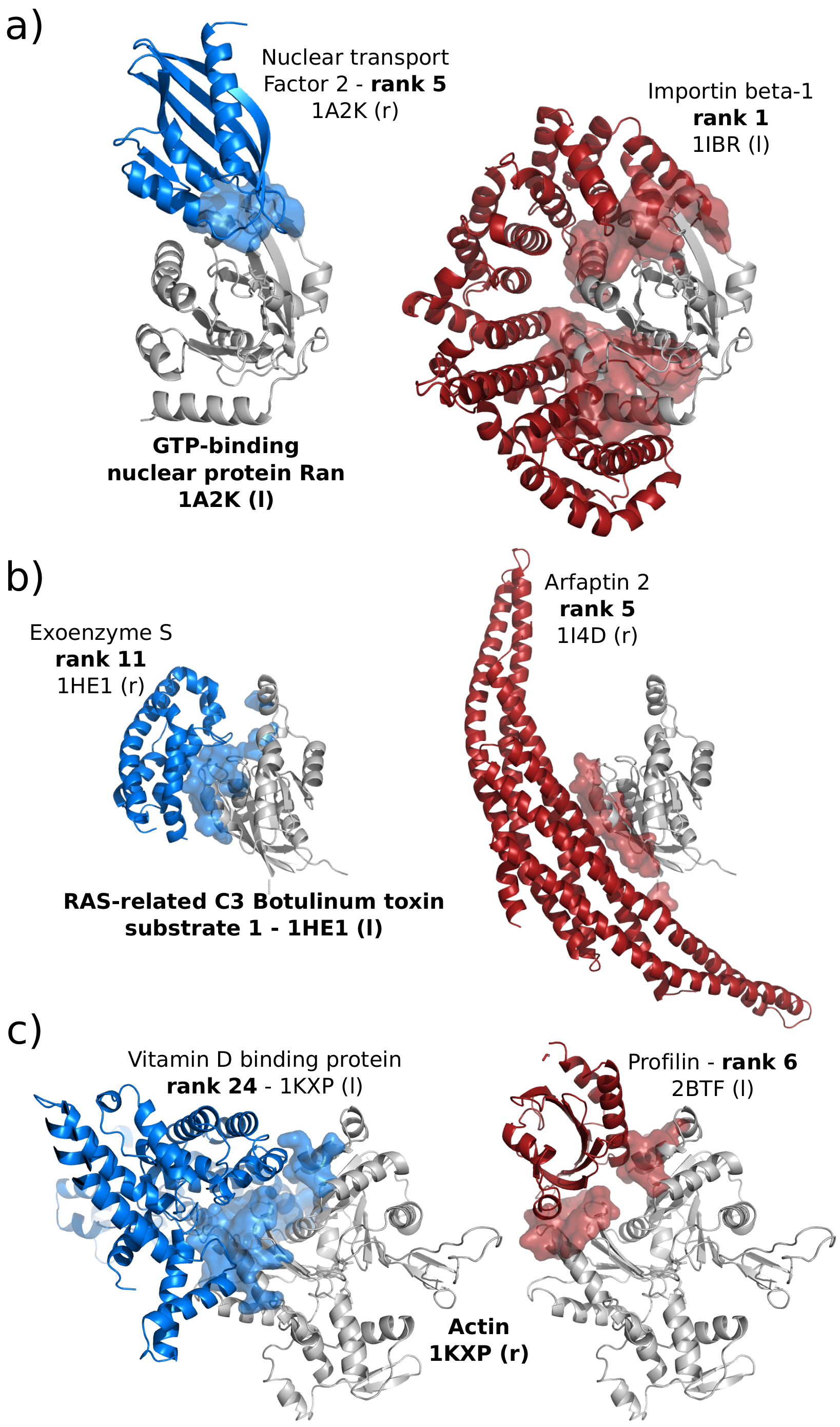

Supplement: S5 Fig — The query protein is represented as a grey cartoon. The cognate partner annotated in the PPDBv2 is shown in blue and a partner identified in the PDB by homology transfer (>90% sequence identity) is shown in dark red. In each case, the proteins come from the same functional class: (A-B) other-with-G protein, OG, (C) others, OX. The intra-class ranks of the partners are given. (TIF) [file pcbi.1009825.s005.tif]

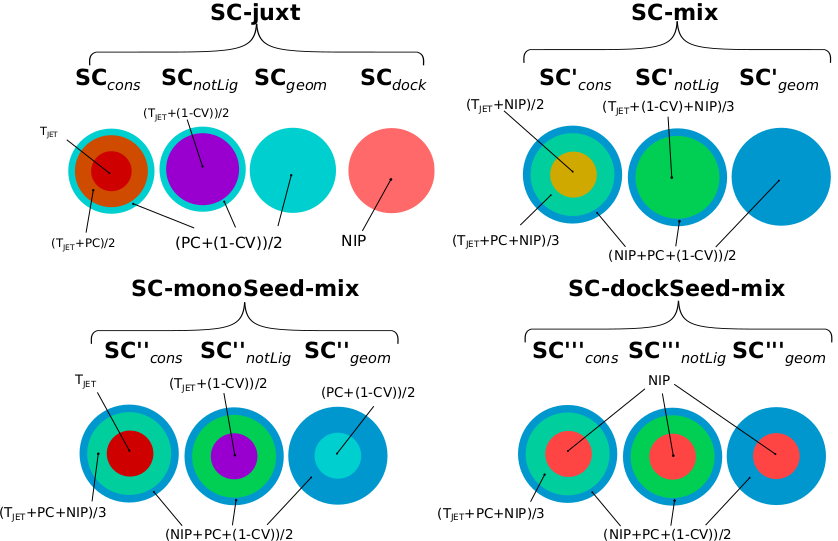

Supplement: S6 Fig — Each scoring scheme is depicted by a schematized representation of a predicted patch, where the different concentric layers correspond to different combinations of four residue-based descriptors. TJET: evolutionary conservation. PC: physico-chemical properties. CV: circular variance. NIP: docking-inferred binding propensities. Top left panel: SC-juxt comprises four scoring schemes, three of them (SCcons, SCnotLig and SCgeom) using TJET, PC and CV and the fourth one (SCNIP) exclusively based on NIP. SCcons detects highly conserved seeds and extend them using physico-chemical properties and local geometry. SCnotLig is a variant of SCcons including circular variance at the seed detection step to avoid buried ligand-binding pockets. SCgeom disregards evolutionary conservation and detects protruding regions with good phyisco-chemical properties. All other scoring schemes are variants of SCcons, SCnotLig and SCgeom including NIP in different ways. Top right panel: SC-mix combines NIP with the other descriptors at each step. Bottom left panel: SC-monoSeed-mix disregards NIP to detect the seeds and then combines it with the other descriptors. Bottom right panel: SC-dockSeed-mix relies exclusively on NIP to detect seeds and then uses a combination of the four descriptors. (TIF) [file pcbi.1009825.s006.tif]

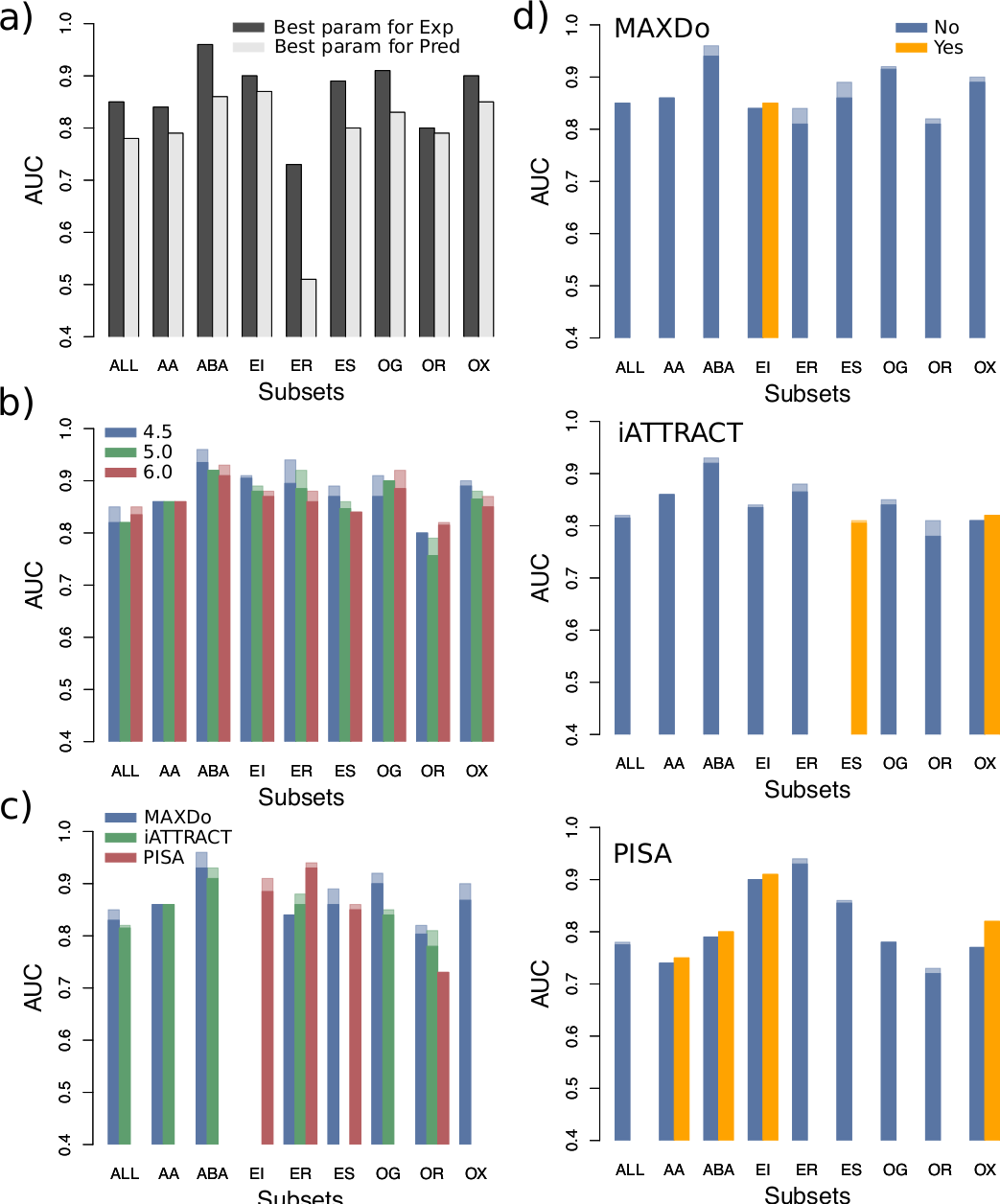

Supplement: S7 Fig — (A) Comparison of the AUC values obtained when the parameters were optimized for dealing with experimental interfaces or for dealing with predicted interfaces. The parameters for experimental interfaces are a 6 Å threshold, the MAXDo energy function and no CIPS. They were applied to all classes but EI, where PISA was used instead of MAXDo. The parameters for predicted interfaces are a 5 Å threshold, the MAXDo energy function and CIPS. There are three exceptions: PISA was used for EI, iATTRACT was used for ER and CIPS was not used for OR. (B-D) Influence of the individual parameters on the predictive performance. (B) Distance threshold used to define docked interfaces. (C) Docking energy. (D) Presence or absence of the CIPS pair potential, depending of the docking energy. In each plot, for each protein class, we considered the 6 combinations with the highest AUC values. This pool of combinations was divided into 2 to 4 subsets depending on the number of values considered for the parameter. The opaque bars indicate the average AUC values computed over the subsets of combinations. The parts in transparent indicate the maximum values. If a parameter value was not present in the 6 best combinations, then it does not appear on the plot. (TIF) [file pcbi.1009825.s007.tif]

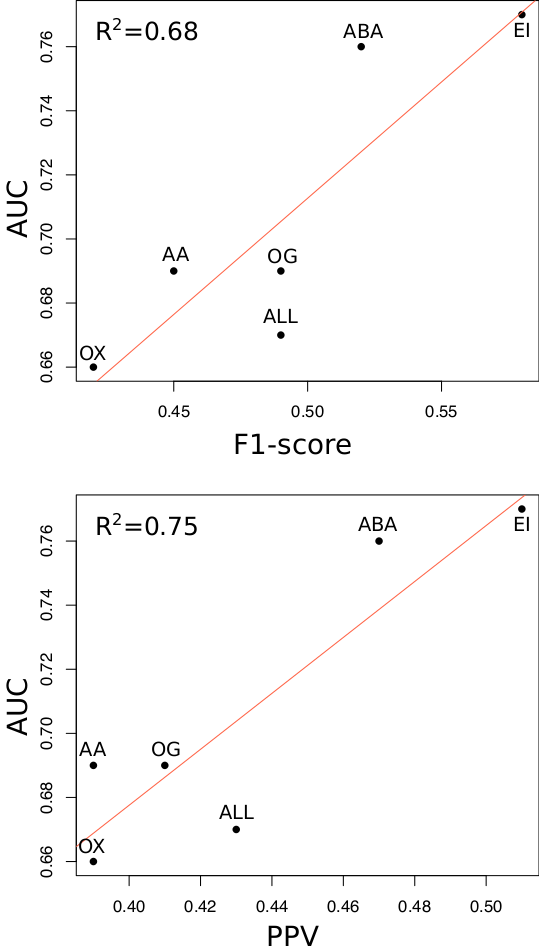

Supplement: S8 Fig — The AUC values are plotted in function of the F1-score and the positive predictive value (PPV) of the predicted RIs, for the whole dataset and a subset of classes (each containing more than 15 proteins). On each plot, the red line corresponds to a linear regression between the two variables, whose adjusted R2 is reported in the top left corner. The scoring strategy is SC-dockSeed-mix and the AUC values correspond to CCD2PI default parameter setting. (TIF) [file pcbi.1009825.s008.tif]

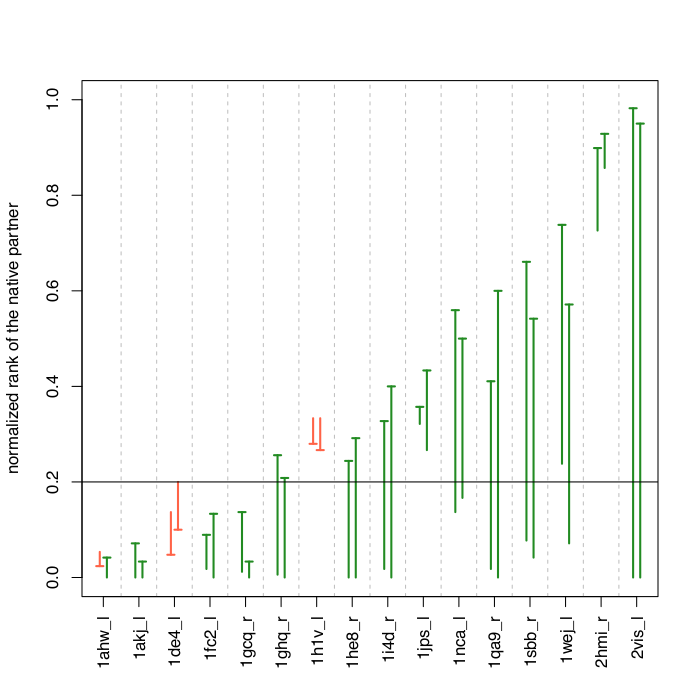

Supplement: S9 Fig — For each protein, we show the improvement (in green) or the deterioration (in red) of the native partner’s rank upon replacing the predicted RIs with the experimental interfaces. The ranks obtained using the predicted RIs are marked with horizontal ticks—the other extremity of the segment corresponding to using the experimental RIs. The partner is identified either within the whole PPDBv2 (left segment) or only within the functional class of the protein (right segment). (TIF) [file pcbi.1009825.s009.tif]

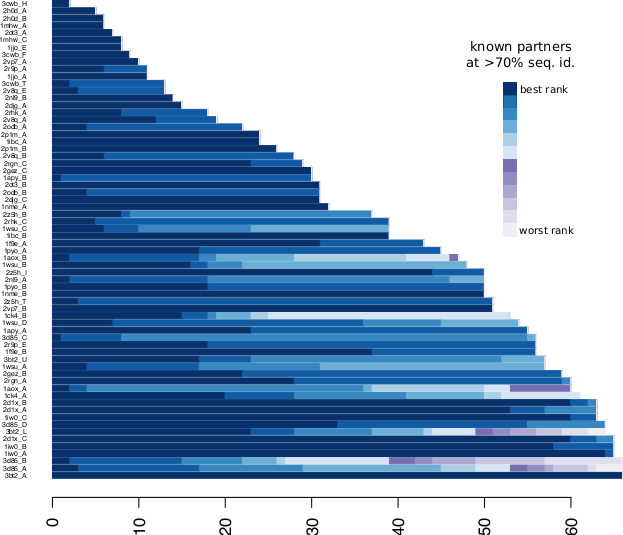

Supplement: S10 Fig — For each protein from the set, the barplot indicates the rank(s) determined by CCD2PI for its known partner(s). The partners are inferred from the complex PDB structures involving the proteins from the set or their close homologs, sharing more than 70% sequence identity. There are up to 12 partners for each protein, and they can be distinguished by the blue and purples tones. Compare with Fig 5A in the main text. (TIF) [file pcbi.1009825.s010.tif]

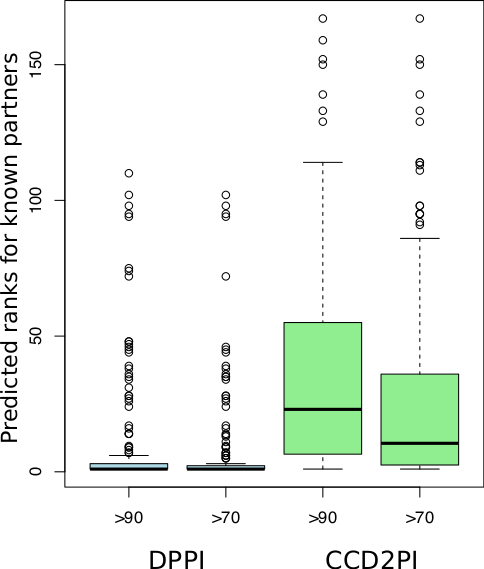

Supplement: S11 Fig — Distributions of the best ranks predicted by DPPI (left, lightblue) and CCD2PI (right, lightgreen) for the known partners, inferred at 90 and 70% sequence identity levels. (TIF) [file pcbi.1009825.s011.tif]
