# Supplementary material for: From complete cross-docking to partners identification and binding sites predictions
Source: PLoS Comput Biol. 2022 Jan 28;18(1):e1009825. doi: 10.1371/journal.pcbi.1009825 (PMC8827487; doi:10.1371/journal.pcbi.1009825)
Supplement: Attachment — Submitted filename: response2Reviewers.pdf [file pcbi.1009825.s012.pdf]

Reviewer #1:

Dequeker et al. present a protein-protein cross docking approach to identify binding partners for a given set of proteins. It is based on previous work by the Carbone group and indicates significant improvements. Prediction of potential binding partners of proteins is one major goal of bioinformatics and computational biology. The route taken by the authors is interesting and differs from other approaches (mostly based on machine learning trained by experimental input data). The prediction is based on cross docking of all against all proteins in the data set (structures of the proteins need to be known). The approach has the potential to identify protein-protein interaction pairs that cannot be detected based on homology to some known interacting pair.

We thank the reviewer for his positive comments.

I have, however, some comments:

1. Although the approach is quite clearly explained the scoring and selection of correct complexes is sometimes difficult to understand. As a scoring function the authors use a product of overlap of the interface of a docked structure and the Energy of the docked complex and the pair potential energy. It is not clear why the authors use here a product (as interaction index) and not for example the sum of these contributions (appropriatly weighted).

By formulating the score as a product, we effectively use the interface overlap, the docking energy and the pair potential as successive filters to select the best conformation. The rationale is that ideally, the selected conformation should meet all three criteria: match the expected interface, be energetically favourable, and reflect the amino-acid pairing preferences found in experimental complexes. For instance, let us consider a conformation displaying a perfect interface overlap, but with the interacting surface of the ligand rotated by 180° with respect to that of the receptor. It would have a very low fraction of native contacts, and we expect it to be correctly filtered out by the docking energy and/or the pair potential. We added some sentences in the subsection "Computational framework" of the Results section on p. 6 to clarify this point. Following the reviewer suggestion and using a weighted sum instead of a product could help to limit the impact of approximations in the predicted reference interfaces on partner discrimination. We now discuss this possibility in the Discussion part on p. 14.

2. In the scoring the authors use a predicted "reference interface" (not the real native interface) for comparison with a docking interface. This type of reference interface may differ from the correct "native interface". Did the authors check how often this is the case? In such case an incorrect structure would be favored by the score!

Among the 168 proteins in the PPDBv2 set, 16 display predicted interfaces that do not match with the experimental ones (very low F1-score, below 0.2). In half of the cases, we found that replacing the predicted interfaces by the experimental ones largely improves the ability to single out the cognate partner (see Figure 1 below). Nevertheless, in the remaining half, improving interface quality brings little gain to partner identification, or even has a deleterious impact. In five cases, the cognate partner is even identified in the top 20% despite the low quality of the predicted RI. These results reveal the existence of protein surface regions onto which cognate partners bind more favourably than non-interactors, although they have not been experimentally characterised as directly involved in the interaction. We hypothesise that these regions might correspond to alternative binding modes with the cognate partner. Our scoring strategy might provide a mean to unravel such alternative binding modes.

We added these results in the subsection " Small approximations in the reference interfaces may significantly impact partner identification" of the Results section, on p.12. We also added a sentence in the discussion on p. 15.

3. The authors identify the "correct" partner for a protein with reasonable accuracy (AUC 0.67) and

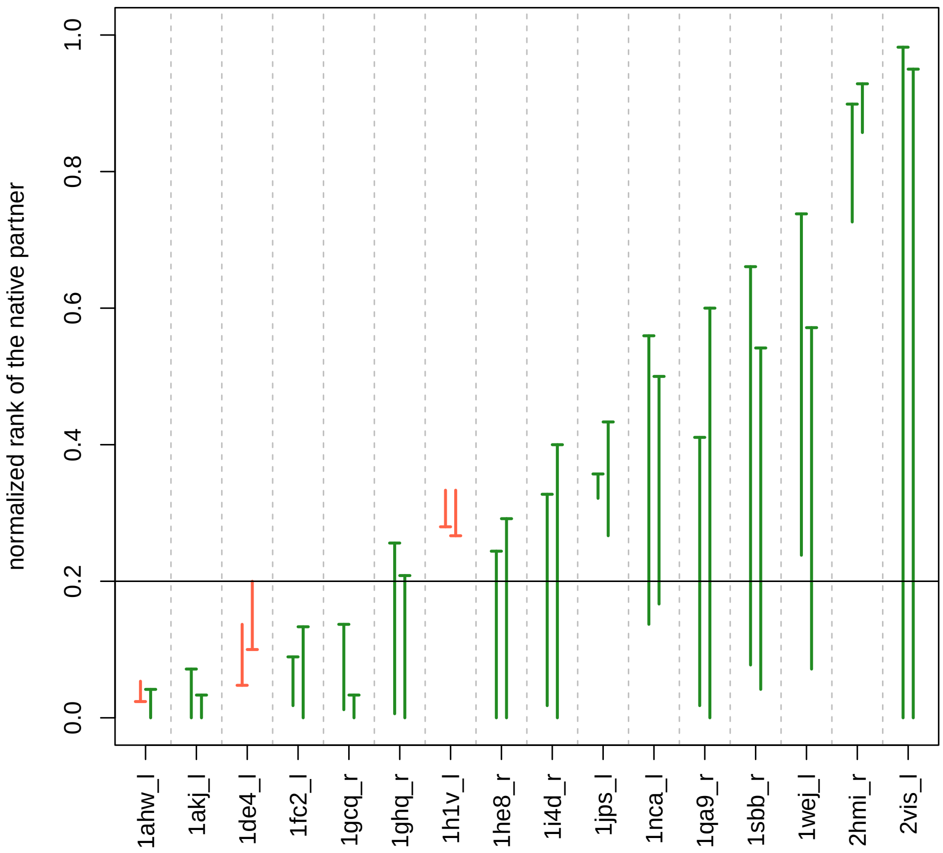

Figure 1: **Partner identification for proteins where the predicted and experimental interfaces do not match (F1-score $<$ 0.2).** For each protein, we show the improvement (in green) or the deterioration (in red) of the native partner's rank upon replacing the predicted RIs with the experimental interfaces. The ranks obtained using the predicted RIs are marked with horizontal ticks – the other extremity of the segment corresponding to using the experimental RIs. The partner is identified either within the whole PPDBv2 (left segment) or only within the functional class of the protein (right segment).

claim that there are only few predicted alternative high affinity (incorrect) complexes. However, my impression by looking at Fig. 2 is that there are quite a number of ”dark” spots in the interaction matrix such that there are something like 10-20 putative PPIs for each given protein (on average)?

On average, 3 putative partners are predicted with a NII score above 0.8, and about 10 above 0.6, for each given protein (see Figure 2 below). We added a sentence in the subsection ”CCD2PI accurately singles out cognate partners within specific functional classes” of the Results section on p.8, and a supplementary figure, numbered S1.

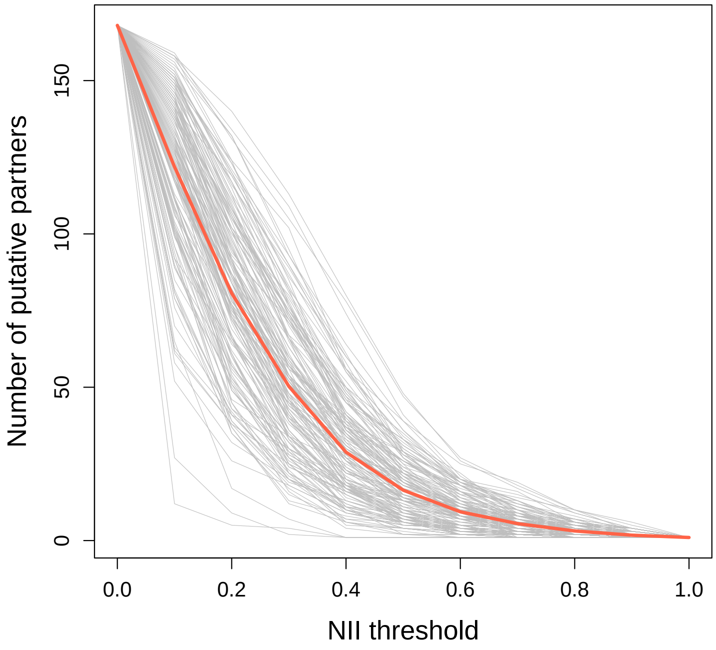

Figure 2: **Number of putative partners predicted by CCD2PI.** Each grey curve corresponds to a protein from the PPDBv2, and indicates the number of putative partners (y-value) with a NII greater than a threshold (x-value). The red curve shows the average behaviour.

4. I think it could be useful to look in more detail at some of the "incorrect" predictions. What is the main reason that some incorrect predicted complexes get high interaction scores and hence would be predicted as forming a realistic complex? This is not dicussed.

We thank the reviewer for pointing this out. In principle, the docking energy and the pair potential in Eq. 1 (II formula) should favour the protein pairs whose RIs have a high physico-chemical and shape complementarity. Consistently, we observed that the RIs of the proteins predicted as plausible partners for a given protein share some common 3D physico-chemical patterns. For instance, we can clearly identify a pattern of positively charged residues common to the RIs of the "incorrect" top 5 predicted partners for the human GTPase-activating protein p120GAP (1WQ1_l) and the RI of its cognate partner H-RAS, ranked at the 6th position (see Figure 3 below, panel A). In the case of the human gonadotrophin (1QFW_l), the RI of its cognate antibody, ranked 13th, displays an enrichment in negatively charged and aromatic residues, also observed for the RIs of the "incorrect" top 5 predicted partners (see Figure 3 below, panel B). We added these results in the subsection "CCD2PI accurately singles out cognate partners within specific functional classes" of the Results section on p.8, and we added the figure 3 below in the supplementary material.

5. The approach is very computationally demanding which could be a major drawback in putaitve applications. The authors should extend the discussion section to indicate possible sets of proteins (containing a limited number of partners) for which an application of the approach could be useful.

We agree that complete cross-docking calculations between hundreds of proteins remain computationally demanding. Nevertheless, they can be efficiently parallelized on grid-computing systems. Here, the docking calculations were distributed on the public World Community Grid. For a more convenient usage on a personal computer, the approach can be applied to discover and characterise interactions between proteins involved in a particular metabolic or signaling pathway. For instance, one could use it to explore the inter-

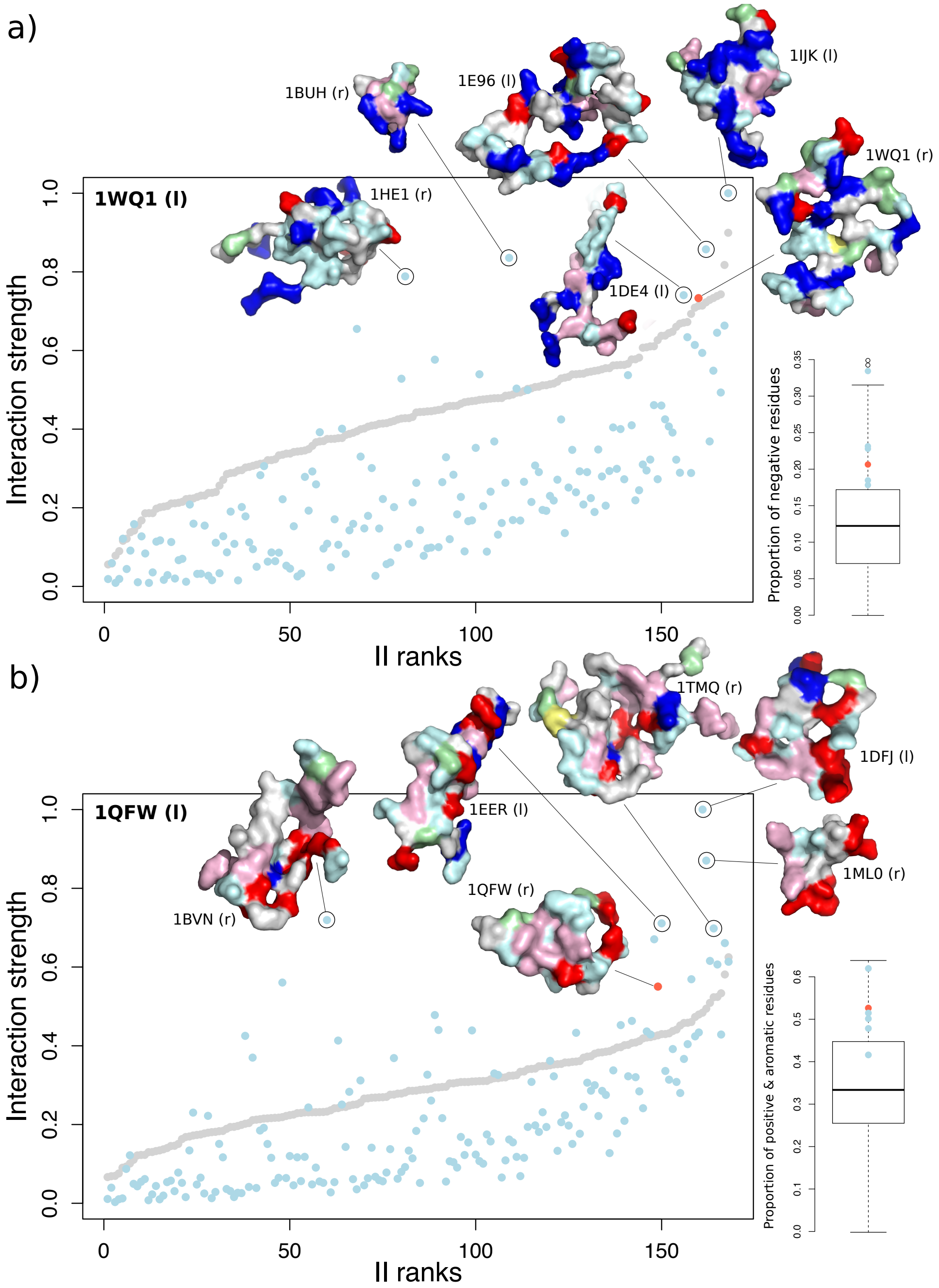

Figure 3: **Comparison of cognate partners and competitors interfaces.** The "interaction strength" is used for plotting pairs with respect to II values (grey) and NII values (blue). The 168 proteins are ordered along the x-axis according to the II ranks they obtained with the protein of interest, and for each position on the x-axis, two points are plotted. The point corresponding to the NII value of the cognate partner is highlighted in red. The II values are scaled between 0 and 1. The predicted RIs for the cognate partner and the top 5 competitors are depicted as surfaces colored by amino acid properties: positive (KR) in blue, negative (DE) in red, polar (HNQST) in cyan, aromatic (FWY) in pink, hydrophobic (AGILMV) in white, cysteine (C) in yellow, and proline (P) in green. The boxplots show the distribution of the proportion of positives (panel a) or negatives and aromatic (panel b) residues in the RIs. The values for the cognate partner and the top 5 competitors are indicated by colored dots.

actions between the 11 enzymes of the Calvin-Benson cycle and their inhibitors/activators. We extended the Discussion section on p. 16 to mention these practical aspects.

Reviewer #2:

Prediction of protein-protein interaction networks is one of the most important biological problems. Experimental techniques for determination of such networks, based on high-throughput methodologies are known to be not very accurate. Computational approaches based on sequence information have been around for a long time, and also suffer from limited accuracy. This study continues the authors' series of publications addressing this issue from the perspective of structural modeling, which is a useful complement to the experimental and sequence-based techniques. In this study, the authors added more structural and physicochemical parameters to their previously used set. The new, expanded set showed a significantly better discrimination power to the non-cognate interactors, thus considerably improving the utility of the method. The paper contains detailed analysis of the approach, parameters variation, and performance on the general and function-specific protein-protein sets, as well as comparison with alternative approaches. The approach is a useful addition to the arsenal of computational techniques for characterization of protein interactions, and as such would be of interest to the biological community.

We are grateful to the reviewer for his positive comments.

Reviewer #3:

The authors propose a molecular docking approach to predict protein partners and interaction strength. They assessed their algorithm for recovery of known and predicted protein-protein interactions, finding that their algorithm improves upon previous partner identification methods, although it is outperformed by a deep learning method.

We thank the reviewer for her/his comment.

MAJOR CONCERNS

The pair extension by homology is fundamental to the paper, but the authors do not provide sufficient evidence for its validity, or for the thresholds used. The authors acknowledge its limitation, but do not provide sufficient explanation or references. "We considered that a structurally characterized interaction found for P1' and P2', homologs of P1 and P2, respectively, was a strong indicator of the possibility for P1 and P2 to interact with each other. Nevertheless, we should stress that homology transfer does not guarantee that the interaction between P1 and P2 is functional in the cell.

The strategy of transferring the knowledge of protein interactions between close homologs is supported by the observation that functional interfaces are conserved across closely related homologs (Ma *et al.* 2003). Moreover, previous works from us and others have emphasised its biological pertinence and usefulness to evaluate protein-protein/DNA/RNA interface prediction methods (Dequeker *et al.* 2019, Yan *et al.* 2016). We added some sentences in the introduction on p. 4-5 to explicitly refer to these works:

- Ma B, Elkayam T, Wolfson H, Nussinov R. Protein–protein interactions: structurally conserved residues distinguish between binding sites and exposed protein surfaces. Proceedings of the National Academy of Sciences. 2003;100(10):5772–5777.
- Dequeker C, Laine E, Carbone A. Decrypting protein surfaces by combining evolution, geometry, and molecular docking. Proteins: Structure, Function, and Bioinformatics. 2019;87(11):952–965.
- Yan J, Friedrich S, Kurgan L. A comprehensive comparative review of sequence-based predictors of DNA-and RNA-binding residues. Briefings in bioinformatics. 2016;17(1):88–105.

Here, we used high identity cutoffs, namely 90 and 70%, such that we transferred interactions between identical of very similar proteins. These high levels of sequence similarity ensure a high confidence in the newly detected interactions, although homology transfer *per se* does not guarantee they are functional in

the cell. To make this point clearer, we reformulated the sentences pointed out by the reviewer in the "The interaction strengths predicted by CCD2PI reveal the multiplicity of protein interactions" subsection of the Results section on p. 10.

The docking-based partner prediction approach shown here is significantly outperformed by existing machine learning methods (AUC 95% versus 79% recovery of known and inferred partners). The authors are not convincing in the need for their method given the poor performance compared to existing methods.

Indeed, the AUC obtained with the sequence-based deep learning method DPPI is much higher than that obtain with our *ab initio* structure-based approach. Nevertheless, our approach is interpretable and provides a 3D geometrical and physico-chemical description of protein interacting surfaces that can serve as a useful complement to the sequence-based predictions. To illustrate this point, we added a readout of the different contributions in the II and NII formulas, taking two proteins as illustrative examples.

The docking energy and the pair potential in the II formula will favour the protein pairs whose RIs have a high physico-chemical and shape complementarity. Consistently, we observed that the RIs of the proteins predicted as plausible partners for a given protein share some common 3D physico-chemical patterns. For instance, we can clearly identify a pattern of positively charged residues common to the RIs of the "incorrect" top 5 predicted partners for the human GTPase-activating protein p120GAP (1WQ1 l) and the RI of its cognate partner H-RAS, ranked at the 6th position (see Figure 4 below, panel a). In the case of the human gonadotrophin (1QFW l), the RI of its cognate antibody, ranked 13th, displays an enrichment in negatively charged and aromatic residues, also observed for the RIs of the "incorrect" top 5 predicted partners (see Figure 4 below, panel b). This analysis helps us to rationalise the "incorrect" predictions.

Another important ingredient in our approach is the normalisation of the interaction indexes. Specifically, by lowering down the interaction strengths computed for highly sociable proteins, the normalisation eliminates most of the "incorrect" partners. Given a protein, only the putative partners binding favourably to it, with a high II score, and in a specific manner, as indicated by a low S-index, stand out after the normalisation. This effect is clearly visible on the two above-mentioned examples (see Figure 4 below). A systematic analysis of the effect of the sociability-based normalisation on different parts of the interface could give clues about the specificity determinants of molecular recognition.

We added these results in the subsection "CCD2PI accurately singles out cognate partners within specific functional classes" of the Results section on p.8, and we added the figure 4 below in the supplementary material. We also added some sentences in the Discussion section on p. 14,16.

Authors use different levels of homology to define true pairs between different datasets and figures (Figure 2, 5).

We thank the reviewer for pointing this out. The barplot in Figure 5a indeed shows only the results corresponding to the 90% sequence identity level. We added a supplementary figure, numbered S10, to show the results corresponding to the 70% sequence identity level. The inset in Figure 5a reports the results for the two levels, as Figure 2b.

The probability at random (grey bars) in Figure 2b is unclear.

In Figure 2b, we report the proportion of proteins with at least one known partner in the top X% predictions, computed as:

$$P_{topX} = \frac{1}{N}\sum_{i=1}^{N} \mathbb{1}_{\min_k(R_k^i) \leq \frac{X}{100}N}, \tag{1}$$

where R_k^i is the rank of the kth partner predicted by CCD2PI for protein P_i, and N is the total number of proteins in the set. We put this proportion in context with respect to some baseline value computed by

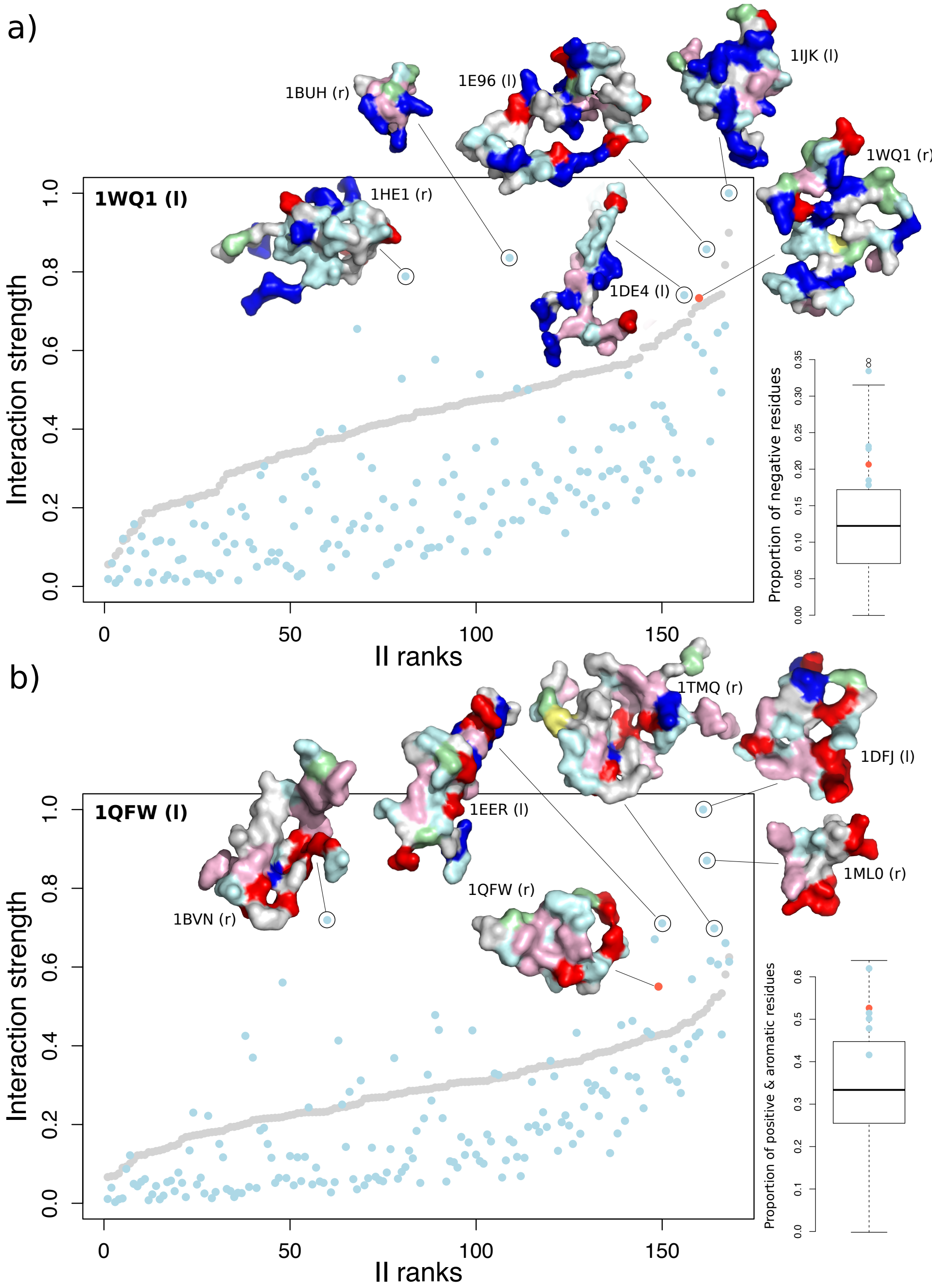

Figure 4: **Comparison of cognate partners and competitors interfaces.** The "interaction strength" is used for plotting pairs with respect to II values (grey) and NII values (blue). The 168 proteins are ordered along the x-axis according to the II ranks they obtained with the protein of interest, and for each position on the x-axis, two points are plotted. The point corresponding to the NII value of the cognate partner is highlighted in red. The II values are scaled between 0 and 1. The predicted RIs for the cognate partner and the top 5 competitors are depicted as surfaces colored by amino acid properties: positive (KR) in blue, negative (DE) in red, polar (HNQST) in cyan, aromatic (FWY) in pink, hydrophobic (AGILMV) in white, cysteine (C) in yellow, and proline (P) in green. The boxplots show the distribution of the proportion of positives (panel a) or negatives and aromatic (panel b) residues in the RIs. The values for the cognate partner and the top 5 competitors are indicated by colored dots.

counting the number of times we expect to find at least one known partner in a randomly chosen subset:

$$P_{topX}^{base} = \frac{1}{N}\sum_{i=1}^{N} \mathbb{1}_{\frac{X}{100}N_i \geq 1}, \tag{2}$$

where N_i is the number of known partners for protein P_i. The grey bars correspond to the baseline values. We added a subsection "Assessment of the predictions" in the Materials and Methods section on p. 20 to clarify the content of figure 2b. We now refer to this subsection in the legend of the figure.

Figure 5a inset is unclear, what are the grey bars? Additionally, this figure does not clearly support "CD2PI identifies at least one known partner in the top 3 for about a third of the 319 proteins (Fig. 5a, inset)"

The information given in the inset of Figure 5a is similar to that of Figure 2b. We rewrote the legend of the inset to make it more clear. We added an explicit mention of the gey bars. The inset shows that CCD2PI identifies at least one known partner in the top 5%, in other words the top $5 \times 62/100 = 3$ predictions, for about a third of the proteins. The fact that 3 proteins correspond to 5% of the set is now explicitly written in the subsection "Accounting for protein surface multiple usage" of the Results section on p. 13.

Figure 5a is unclear. What are the structures shown in colors? Caption describes these structures as known partners, but there are not 6 blue tones for 2r9p:A.

We thank the reviewer for indicating us this problem. We fixed the legend of Figure 5a to clearly indicate what the depicted 3D structures are. The known partners are in blue and the incorrect partners with better predicted ranks are in other colors. We also changed the way we describe the figure in the main text.

Figure 5b should include all proteins, not just cases where CCD2PI outperforms DPPI

Our goal with Figure 5b was to emphasise some potentially interesting examples where CCD2PI outperforms DPPI. Including all proteins would prevent the clear identification of these cases. To show the full distributions of ranks predicted for the known partners, we added a supplementary figure, numbered S11, and we now refer to it in the legend of Figure 5b.

MINOR POINT
"We combine together physics-based energy, interface matching and protein sociability, three ingredients we previously showed to be relevant to partner identification and discrimination." Lacks citation.

We added some references to our previous works:

- Laine E, Carbone A. Protein social behavior makes a stronger signal for partner identification than surface geometry. Proteins. 2017 Jan;85(1):137–154.
- Lopes A, Sacquin-Mora S, Dimitrova V, Laine E, Ponty Y, Carbone A. Protein-protein inter- actions in a crowded environment: an analysis via cross-docking simulations and evolutionary information. PLoS computational biology. 2013;9(12).
- Sacquin-Mora S, Carbone A, Lavery R. Identification of protein interaction partners and protein- protein interaction sites. J Mol Biol. 2008;382:1276–1289.

In summary, some figures in the paper are somewhat difficult to interpret. The evidence supporting pair extension by homology is moderate, and the approach is significantly outperformed by existing methods.

We modified the legends of Figure 2 and 5 and added some explanations about the calculation of the expected baseline values in the main text. We modified Figure 5, and complemented it with two supplementary figures (S10 and S11). We added a motivation and some references for the homology transfer of interactions. We added a detailed analysis of a couple of proteins, with a new supplementary figure (S3), to illustrate the contributions of the different terms in our calculation of the interaction strengths. We showed

that the ”incorrect” predictions display realistic features and might actually correspond to unknown partners and binding modes. We added some sentences in the discussion to emphasise the utility and applicability of the proposed approach. We hope that these elements have improved the clarity of the manuscript and have underlined the interest of the approach we propose.
