# Supplementary material for: From complete cross-docking to partners identification and binding sites predictions
Source: PLoS Comput Biol. 2022 Jan 28;18(1):e1009825. doi: 10.1371/journal.pcbi.1009825 (PMC8827487; doi:10.1371/journal.pcbi.1009825)
Supplement: Attachment — Submitted filename: Response-to-reviewer-3.pdf [file pcbi.1009825.s013.pdf]

Reviewer's Responses to Questions

Comments to the Authors:
Please note here if the review is uploaded as an attachment.

Reviewer #1: I carefully read the new version of the manuscript and the responses to my concerns. I think the authors successfully responded to my concerns. The manuscript is an interesting approach to predict new putative protein-protein interactions. Very recently, AI approaches such as Alphafold2 have become very successful in predicting not only protein structures but also putative protein-protein interactions. I think it could be very valuable for the manuscript to relate/discuss the "docking" approach of the authors vs. emerging AI based approaches to predict complex structure. This could further improve the manuscript.

We would like to thank the referee for the suggestion. We have addressed it by adding a new paragraph in the discussion on the "docking" approach reported in this manuscript vs. AlphaFold2.

Have the authors made all data and (if applicable) computational code underlying the findings in their manuscript fully available?
The PLOS Data policy requires authors to make all data and code underlying the findings described in their manuscript fully available without restriction, with rare exception (please refer to the Data Availability Statement in the manuscript PDF file). The data and code should be provided as part of the manuscript or its supporting information, or deposited to a public repository. For example, in addition to summary statistics, the data points behind means, medians and variance measures should be available. If there are restrictions on publicly sharing data or code —e.g. participant privacy or use of data from a third party—those must be specified.

Reviewer #1: Yes

PLOS authors have the option to publish the peer review history of their article (what does this mean?). If published, this will include your full peer review and any attached files.

If you choose “no”, your identity will remain anonymous but your review may still be made public.

Do you want your identity to be public for this peer review? For information about this choice, including consent withdrawal, please see our Privacy Policy.

Reviewer #1: No
